# Face to Face with Toy Safety: Understanding an Unexpected Threat

**DOI:** 10.1289/ehp.116-a70

**Published:** 2008-02

**Authors:** Charles W. Schmidt

Until March 2007, thousands of kids around the country could be found playing with toy trucks, helicopters, and soldiers sold under the Elite Operations brand name. The toys were fun, and they looked great with their thick coat of glossy paint. Trouble was, that paint was loaded with 5,000 ppm lead, a potent developmental neurotoxicant with no known safe exposure level.

When the high lead levels were detected during a routine inspection, the Consumer Product Safety Commission (CPSC) issued a recall, the first for a lead-contaminated toy in 2007. Lead-triggered toy recalls were rare, but not unheard of in the United States, with just a handful issued in the last decade. Eventually, nearly 130,000 Elite Operations units—made by a Hong Kong company called Toy Century Industrial and imported by Toys R Us—would be recalled.

In a typical year, the recall would have barely ruffled the $22 billion U.S. toy industry, which sells 3 billion units annually. But 2007 was far from typical as far as import recalls were concerned. Contaminated pet food, cough syrup, toothpaste, and other products—mostly made in China—were being yanked off store shelves under the full glare of the media. Given that most of its wares are made in China, the toy industry ramped up its inspections for lead, and found that high levels were a lot more common than they had assumed. By year’s end, 42 recalls involving nearly 6 million toys had been issued because of excessive lead levels.

Lead-contaminated toys became one of the biggest environmental health stories of recent times. It was shocking to think of children being poisoned while playing, and by lead no less, a toxic metal that consumers assumed had been purged from products long ago. Now lead was back, sparking a furor over toy safety.

## Looking for Answers

“The ‘toxic toy’ issue really exposed holes in safety testing procedures,” says Sally Edwards, a researcher with the Lowell Center for Sustainable Production at the University of Massachusetts Lowell. “The CPSC has responsibility for over fifteen thousand products, but it’s underfunded, understaffed, and dependent on voluntary testing by industry. What’s more, the toy industry is highly competitive; consumers expect low prices, and that forces manufacturers to look for low-cost materials. When you externalize the cost of production, you’re going to pay the price somewhere.”

Years ago, most toys sold in the United States were produced domestically. Now, 87% are produced abroad, according to *Santa’s Sweatshop: “Made in D.C.” with Bad Trade Policy*, a December 2007 report issued by the nonprofit Public Citizen, and of those, 74% are manufactured in China, where it would seem lead paint is used plentifully. A study led by Scott Clark, a professor of environmental health at the University of Cincinnati, found that 50% of the paint sold in China, India, and Malaysia had lead concentrations 30 times higher than the CPSC standard. That finding was published in *Environmental Research* in September 2006.

With manufacturing shifting overseas, U.S. toy importers have come to rely increasingly on test results from foreign suppliers. But overseas testing has been problematic for companies to monitor, and growing evidence suggests it’s more sporadic than one might assume. In congressional testimony given on 19 September 2007, Mattel’s chairman and chief executive officer, Robert A. Eckert, conceded that “a few [overseas] vendors, either deliberately or out of carelessness, circumvented our long-established [testing] standards and procedures.” As a result, Mattel wound up with 3 lead paint–triggered toy recalls in 2007.

Jeff Gearhart, campaign director for the Ecology Center, a nonprofit environmental group in Ann Arbor, Michigan, emphasizes that Chinese toys are not the only culprits. The center’s investigations have shown lead-containing toys originate from numerous countries in addition to China, including Canada, Mexico, Thailand, and the United States. “There’s nothing pristine about the U.S.’s regulatory structure or its production practices that would prevent toxic toys from being produced here,” Gearhart says.

The Ecology Center recently completed the most far-reaching analysis of chemical hazards in toys yet. Their results, published 5 December 2007 on the Consumer Action Guide to Toxic Chemicals in Toys website (www.healthytoys.org), found lead in 35% of 1,200 children’s products tested. Smaller numbers of toys—numbering less than 5% of the total number evaluated—also contained trace amounts of arsenic and/or cadmium. The site now hosts what the Ecology Center says is the most comprehensive public database of toxic hazards in toys in existence, which includes both its own test results and those of other researchers [for more information, see “Consumer Action Guide to Toxic Chemicals in Toys,” p. A69 this issue].

## Unregulated Lead Sources

Among the toys tested by the Ecology Center, 17% had lead concentrations exceeding the CPSC paint standard of 600 ppm. Lead levels in these toys typically ranged from 1,000 to 2,000 ppm. Some of the highest levels weren’t in paint, however, but in vinyl and jewelry, which aren’t regulated by the CPSC. A vinyl Hannah Montana Pop Star Card Game, for instance, contained 3,056 ppm lead.

CPSC spokesperson Julie Vallese says the agency would recall a vinyl toy on account of lead only if children were found to interact with it in ways that could lead to an oral lead dose of at least 175 μg/day. That’s the amount that, according to the agency’s investigations, could cause blood levels to exceed 10 μg/dL, the level at which the Centers for Disease Control and Prevention advises medical intervention. Vallese says that because children typically don’t chew or “mouth” vinyl, the toys aren’t likely to raise blood levels to that concentration, however. Hence, the Hannah Montana Pop Star Card Game can be sold legally, even though its lead content is more than 5 times higher than the enforceable paint standard.

This raises some obvious questions: Are children really less likely to mouth vinyl toys than painted ones? And if they do, will lead leach from vinyl into children’s bodies at rates any different from that at which it leaches from paint? “We don’t find that lead leaches from vinyl,” responds Vallese, adding that the CPSC’s legal mandate—as articulated in the Federal Hazardous Substances Act—requires it to consider exposure in addition to toxicity when evaluating risk; in other words, manufacturers can sell potentially toxic products as long as the exposure pathway is unlikely to be completed.

But Ted Schettler, science director with the Science and Environmental Health Network, a nonprofit group in Ames, Iowa, counters that lead actually can leach from vinyl under conditions that include higher temperatures and low pH. “If a small vinyl toy were swallowed, you can bet the lead would come out; stomach acids would extract it,” he says. Schettler also points to a 25 June 1993 *MMWR Weekly Report* article documenting lead poisoning in a man whose only known exposure was through habitually chewing on lead-impregnated vinyl—in this case, the coating on electrical wires.

Meanwhile, some vinyl toy parts are small enough to swallow. The *Chicago Tribune* on 18 November 2007 reported that vinyl shoes from a Jammin’ Jenna doll made by Ty had lead content averaging 1,980 ppm (however, there is no known case of one of these shoes being consumed).

Vallese responds that an item like a lead-contaminated vinyl shoe, which could possibly be harmful if swallowed, might be subject to additional risk analysis. “We’re working with the Ecology Center now, trying to find out more about the products they analyzed,” Vallese says. “But [apart from paint levels above 600 ppm, which do trigger recalls] we aren’t required to take enforcement action unless the exposure justifies such a measure. We enforce laws, and that’s how the law is written.”

According to Vallese, the CPSC may change its regulations concerning children’s jewelry, which was found by the Ecology Center to contain the highest lead levels of any children’s product on the market. According to the Ecology Center’s investigations, some charms, bracelets, earrings, key chains, rings, and other inexpensive jewelry marketed to children are made entirely of lead. *The New York Times* reported on 29 September 2007 on 2 cases involving children who had swallowed jewelry containing lead. In one, a 4-year-old boy died with blood lead levels of 180 μg/dL after swallowing a heart-shaped charm that came with a pair of Reebok children’s shoes. In another, a 5-year-old girl who ate part of an ankle bracelet was saved by treatment, but not before her blood lead reached 79 μg/dL.

The CPSC acknowledges that children’s jewelry is a problem. “The agency has made it a priority to deal with this issue,” Vallese asserts. “I know kids will put these things in their mouths. We’re trying to get manufacturers to use nonhazardous metals. There’s an exposure risk here that we want to address through the rule-making process.”

Yet even as CPSC’s regulations aim to keep blood lead levels under 10 μg/dL, growing evidence suggests far lower concentrations can produce cognitive problems in children. An investigation by Bruce Lanphear, director of the Cincinnati Children’s Environmental Health Center, which pooled results from 7 studies around the world, found no evidence of a threshold for lead toxicity; IQ impairments that persisted were identified at blood lead levels below 5 μg/dL. Those results were published in the July 2005 issue of *EHP*. “Since then, several studies have confirmed these results,” Lanphear says. “They all found proportionately larger decrements at the lowest levels [of exposure].”

On the basis of these data, the American Academy of Pediatrics recently concluded that the CPSC’s enforceable standard for lead in paint should be dropped from 600 ppm to 40 ppm, which is the upper limit for lead in uncontaminated soil, according to congressional testimony given on 20 September 2007 by Dana Best, an assistant professor of pediatrics at George Washington University School of Medicine.

Vallese says the CPSC is currently bound by law to its existing standard, but pending legislation could change that. A bill passed on 19 December 2007 by the House of Representatives—HR 4040, the Consumer Product Safety Modernization Act, sponsored by Bobby Rush (D–IL)—proposes to gradually reduce the CPSC standard to 100 ppm over 4 years, a level Vallese says would be the strictest in the world.

## Not Just Lead

The lead debacle stunned a toy industry already smarting from ongoing efforts to ban its use of phthalates, vinyl-softening chemicals added to rubber bath toys and teething rings, as well as to cosmetics and medical devices. After more than 50 years of industrial use, phthalates—which cause hormonal changes and reproductive effects in rodents at high doses—can be found in almost all human blood samples from industrialized countries.

Both the toy industry and the CPSC say that phthalates in toys do not put children at risk, claiming that the amounts absorbed by exposure to commercial products are too low to be harmful. Skeptics of that view counter that children’s mouthing behaviors, and also their comparatively more sensitive developing bodies, make them uniquely vulnerable to harm from phthalates and other chemicals. Spurred by activist campaigning, the European Union (EU), the city of San Francisco, and most recently California banned 6 phthalates from children’s products. Both the Toy Industry Association (TIA) and the American Chemistry Council (ACC)—trade groups based in New York and Virginia, respectively—have appealed the San Francisco ban, which is already in effect (the statewide California ban, set to go into effect in 2009, has not been challenged).

It’s not clear how many toys contain phthalates, in part because manufacturers aren’t required to disclose the chemical contents of their products to the public. Sarah Janssen, a scientist at the Natural Resources Defense Council, says soft, flexible bath toys and cosmetics contain some of the highest concentrations and therefore the greatest potential for exposure. Marian Stanley, a senior director at the ACC, says phthalates typically make up 15–20% of the toy’s entire composition. “That’s the amount required for phthalates to do what they do, which is make vinyl soft,” she explains.

According to TIA spokesperson Frank Clarke, toy manufacturers use a single member of this class of chemicals, a compound called di-isononyl phthalate (DINP). Still, studies have found trace amounts of other phthalates in toys. In its own investigation, published on 19 November 2006, the *San Francisco Chronicle* had 16 toys analyzed and found di(2-ethylhexyl) phthalate (DEHP)—a suspected human carcinogen and reproductive toxicant—in a rubber bath toy sold at Walgreens. Other phthalates were also detected, all of them at levels of less than 2%.

Children’s advocates and industry disagree over where the non-DINP phthalates came from. Stanley suggests the reagents and test equipment used during the analysis may have been contaminated with DEHP. Andrew Igrejas, a campaign director with the National Environmental Trust, a Washington, DC–based environmental group, dismisses that view, and insists other phthalates wind up in toys “by mistake” during manufacturing. “It isn’t too farfetched to assume that what this testing reveals is that DEHP continues to be used for some toy applications,” Janssen says. “The source [of the DEHP] should be identified.”

In any case, DINP toxicity is heavily debated. Echoing industry conclusions, the CPSC insists the human risks are nonexistent. In 2002 the agency issued what many cite as the definitive DINP risk assessment. Following that effort, the CPSC performed an extensive exposure assessment, during which mouthing behavior among 169 children aged 3–36 months was recorded by trained observers. DINP “migration” (i.e., leaching) rates from soft plastic toys also were quantified. These measures were used to estimate a maximum daily dose of 2.4 μg of DINP per kg body weight per day. By comparison, the CPSC’s Chronic Hazard Advisory Panel set an acceptable daily intake of 120 μg/kg/day on the basis of histological liver changes in rats, which was the first effect noted.

## Lack of Human Data Breeds Uncertainty

Unfortunately, no comparable data are available on the effect of DINP in humans. Children’s advocates and others who favor phthalate bans typically point to research published in the August 2005 issue of *EHP* by Shanna Swan, a University of Rochester professor of obstetics and gynecology who has shown that phthalate exposure *in utero* is associated with a shortened anogenital distance (the distance from the anus to the base of the penis) in boys aged 2–36 months. These results support findings in male rodents, which show that high-dose phthalate exposures limit the anogenital distance, reduce sperm counts, interfere with testosterone regulation, and impair genital development. However, these findings were based on 9 phthalate metabolites (measured in maternal urine during pregnancy) that Swan concedes are chemically and toxicologically different from DINP.

The whole issue of phthalate toxicity is further complicated by questions surrounding cumulative exposure. Janssen asserts the CPSC’s risk assessment was issued before new evidence of phthalate additivity came to light. Generated in part by Earl Gray, a research biologist at the Environmental Protection Agency, these findings imply that different phthalates act on the same biological pathways such that their effects build on each other. The National Academy of Sciences recently launched a cumulative risk assessment for phthalates, coordinated by project director Ellen Mantus, which is expected to yield a report within 15 months.

In Janssen’s view, the possibility that phthalates may be toxicologically additive further justifies banning them from children’s products. But others insist that doing so will make little or no difference in terms of children’s real-life exposure. Phthalates—produced globally at annual volumes of more than 1 billion pounds—are ubiquitous; indeed the largest source of human exposure is food, according to the Agency for Toxic Substances and Disease Registry.

Two of the most common alternatives to phthalates are acetyl tributyl citrate and DINCH, which is derived from DINP and has a very similar chemical structure. But Stanley counters that while 50 years of use show phthalates to be a relatively sure bet in terms of safety, the alternatives are a roll of the dice. “We don’t know enough about these new plasticizers,” she says. “There still isn’t much data available on them.” To support that position, Stanley cites a 20 April 2000 memo from the CPSC to David Miller, president of the Toy Manufacturers of America (now the TIA), which states that CPSC staff “are concerned that manufacturers not substitute for DINP in children’s products. . . . [E]xisting data are insufficient to determine if acetyl tributyl citrate has any chronic toxic effects that may be relevant to humans.” Stanley confirms the CPSC has no current information on DINP alternatives.

Meanwhile, phthalates have yet to produce a single documented human illness. Schettler concedes we may never know if, or how, early phthalate exposures affect human health. “I don’t know how we could figure that out,” he says. “Animal studies suggest links with reproductive health, but that only becomes manifest when people reach child-bearing age. We’d have to quantify exposures during fetal and early childhood years, and we’d also have to account for other known environmental factors that influence reproductive health—for instance, nutrition.”

Schettler dismisses critics who say it’s unreasonable to remove phthalates from toys if ongoing exposures will still occur from other sources. “My own view is that if you have the opportunity to reduce exposures, then why not do it,” he says. “We do not *need* vinyl toys that kids will mouth.” Ultimately, says Schettler, the decision to avoid phthalates is a precautionary one, based on the notion that it’s better to be safe now than sorry later.

## Proposed Solutions

The European Union invoked the precautionary principle in 2005, when it banned 6 phthalates from children’s products despite objections from its own scientific advisory panel, which felt the documented risks weren’t high enough. In addition to California, 5 other states—Minnesota, Massachusetts, Maine, New York, and Maryland—have introduced legislation to remove phthalates from toys and other children’s products.

With respect to the lead issue, a number of pending bills now aim to boost the CPSC’s power to regulate product testing. Like HR 4040, a Senate bill—SB 2045, sponsored by Mark Pryor (D–AR)—proposes mandatory safety testing (for all relevant elements, not just lead) by third-party inspectors, a measure the CPSC wholeheartedly supports.

Just how the bills will fare in the coming year remains to be seen. President Bush has signaled his support for CPSC reforms, but both he and the agency reject SB 2045’s proposal to make safety violations punishable by a fine of up to $100 million. Vallese emphasizes that a fine of that magnitude would saturate the process with lawyers and inundate the CPSC with paperwork from companies trying to document safety during manufacturing. “We need more safety inspectors, not more attorneys,” Vallese says. The House version proposes a fine of $10 million, which appears to be more palatable to the agency and industry alike.

The CPSC has also begun to address lead paint hazards from imported toys. Whether the amounts in Asian paint have dropped since the toy recalls started last year is unknown. According to Vallese, the CPSC is addressing that issue now. “We need to deal with the problem at its source,” she asserts. “So we’ve entered into agreements with the Chinese government to address safety in production; we signed those agreements in September [2007].” [For more information on these agreements, see box insert this page.]

For parents, lead and phthalate avoidance is easier said than done, given that the chemical components of toys are not usually made publicly known. Gearhart emphasizes that cheap jewelry should be avoided at all cost. Parents can search healthytoys.org, where test results on specific toys are posted as they emerge. Toys made with nontoxic paints and materials present another increasingly widespread option. Ultimately, though, the toy recalls of 2007 are in some ways more a wakeup call for industry and federal regulation than a trigger for excessive parental anxiety. Over time, they are certain to spur some beneficial changes.

## CPSC: In Search of Safety

The extraordinary number of lead-contaminated toy recalls in 2007 has put the Consumer Product Safety Commission (CPSC) under growing public scrutiny. The CPSC’s primary mandate is to help industry develop voluntary safety standards and to issue mandatory standards when the agency deems those produced voluntarily by industry to be insufficiently protective. But the CPSC is also directed by Congress to conduct routine product inspections to ensure that harmful wares don’t reach the marketplace.

Don Mays, senior director for product safety at the Consumers Union (CU), the nonprofit publisher of *Consumer Reports*, says there are just 15 CPSC inspectors monitoring all 300 ports in the United States (the agency has traditionally rotated from port to port, making its presence at any given location intermittent). The CPSC has traditionally not measured for chemical exceedances at the borders, leaving that responsibility with importers, who are liable for any harm caused by products they sell.

Thanks in part to a dwindling budget—which has not kept pace with annual inflation—the CPSC’s full-time staff has fallen from a high of 890 in 1973 to roughly 400 today, according to Martin Bennett, a retired CPSC inspector. Martin says the number of field inspectors has fallen due to staff attrition, a point that CPSC spokesperson Julie Vallese affirms is true. Advocacy groups assert that staff losses have severely diminished the CPSC’s ability to keep up with rising imports from global trade. “They just don’t have the resources they need to keep up with screening,” says CU spokesperson Ami Gadhia.

For fiscal year 2008, Congress added $17 million to the CPSC’s 2007 budget of $63 million, the first real increase since 1981, Vallese says. Some of that money will be used to hire border inspectors and to purchase 10 handheld X-ray fluorescence devices at roughly $30,000 apiece. These devices are used to analyze the chemical content of products.

The CPSC has also initiated new measures to boost port inspections. A newly expanded Import Surveillance Division, announced on 7 January 2008, will establish a tracking system at ports of entry throughout the United States. The system will generate real-time information about U.S.-bound shipments even before they leave foreign ports. Although the system will bolster efforts to ensure product safety, Mays points out that full-time staff will be posted at only 2 ports (Long Beach and Seattle). Moreover, the tracking system will not be operational until 2011, he says.

Vallese emphasizes the real thrust of the CPSC’s expanded efforts to block hazardous toys from the market won’t take place at the borders or the ports. “We have to go to the source,” she says. Along those lines, the CPSC has been holding ongoing meetings with representatives from the Chinese government. In agreements signed in September 2007, the General Administration of Quality Supervision, Inspection, and Quarantine of the People’s Republic of China, which is the CPSC’s regulatory counterpart in China, agreed to ensure that Chinese manufacturers adhere to U.S. safety standards, Vallese says. They also created a paint certification system that guarantees paint lead levels meet CPSC safety standards and agreed that manufacturers who violate safety standards will be stripped of their export licenses.

Mays says the CPSC has signed similar agreements with at least 10 other countries. Most of these agreements were signed before the dramatic rise in lead paint–related recalls began during 2007. “The bottom line is that the CPSC needs more port inspectors,” he says. “And they have to start levying fines against violators.” As it currently stands, the CPSC is authorized to fine those who violate safety standards up to $1.8 million. According to Mays, none of the toy importers subjected to lead-related recalls were fined. **–Charles W. Schmidt**

## Figures and Tables

**Figure:**
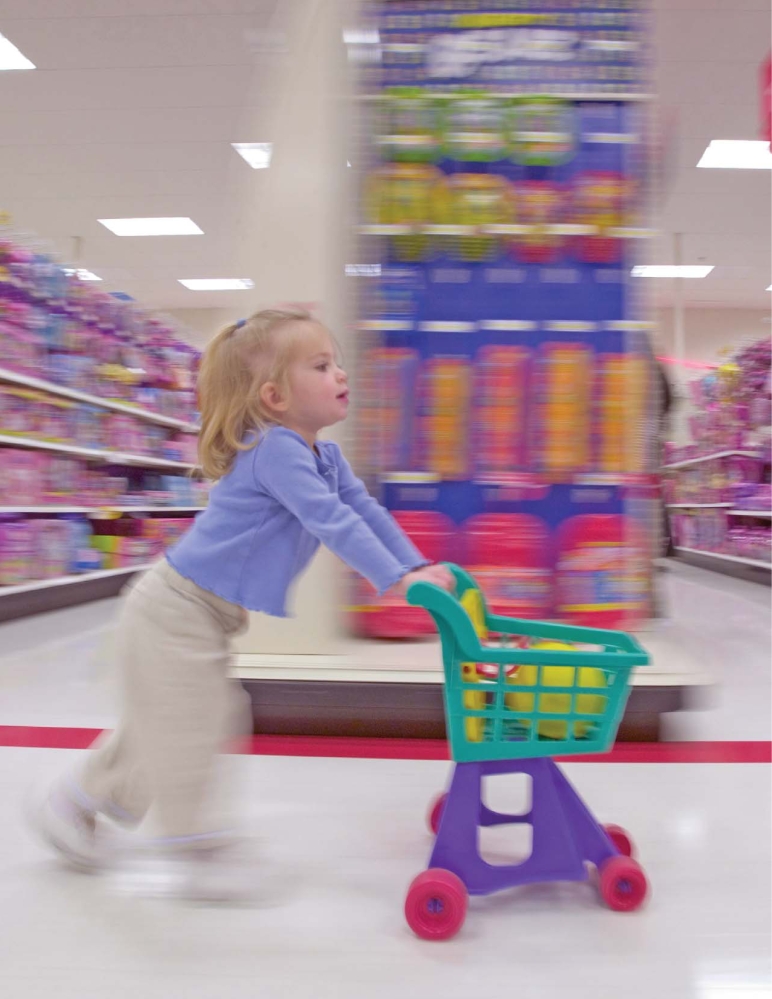


**Figure:**
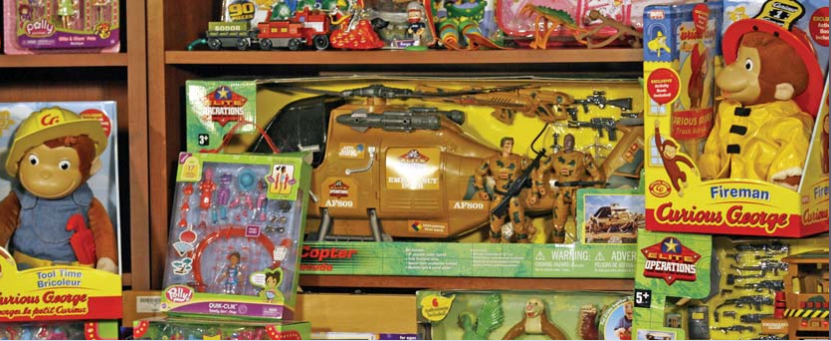
The $22 billion U.S. toy industry sells about 3 billion toys each year. In 2007 there were 81 toy recalls for a variety of reasons. Half of these, involving nearly 6 million toys, were related to lead paint.

**Figure:**
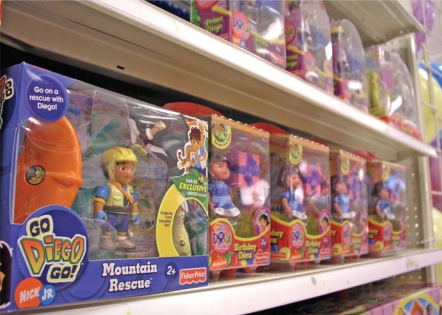
Who can you trust? Ramped-up safety inspections in recent months revealed that even trusted brands of toys could contain potentially unsafe levels of lead. Many experts cite the shifting of manufacturing overseas—which makes monitoring more difficult—as a reason why hazardous materials are turning up in consumer products.

**Figure:**
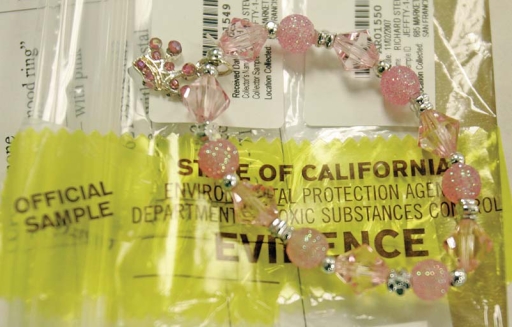
High-risk adornment This child’s bracelet was found by the California Environmental Protection Agency’s Department of Toxic Substances Control laboratory to contain unsafe levels of lead. One-third of the children’s jewelry tested so far by the California Department of Toxic Substances Control contained excessive levels of lead. Moreover, studies by the Ecology Center have shown jewelry to contain some of the highest lead content of all children’s products tested.

**Figure:**
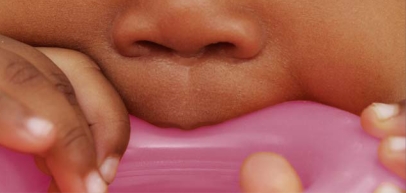
Both the toy industry and the CPSC say that phthalates in toys do not put children at risk, but skeptics counter that children’s mouthing behaviors make them uniquely vulnerable to harm from these chemicals. The European Union, the state of California, and the city of San Francisco have banned 6 phthalates from toys largely on a precautionary basis.

**Figure:**
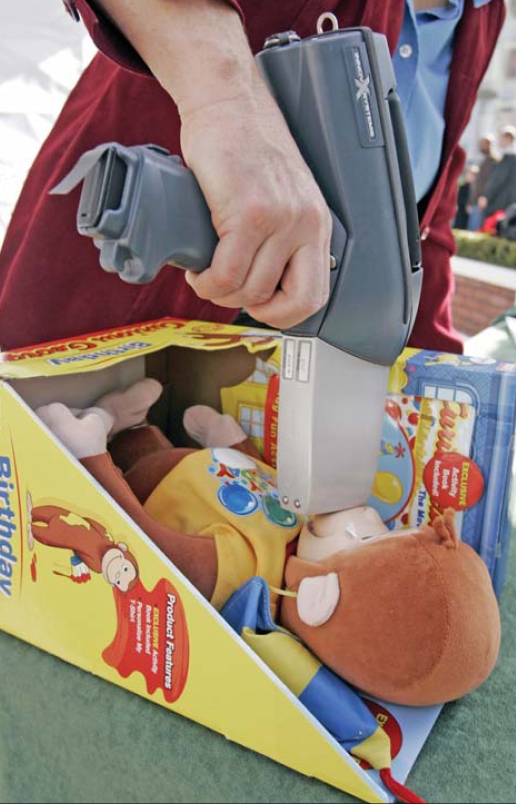
The exposure variable A portable X-ray fluorescence analyzer can ascertain content of toxicants such as lead, arsenic, and cadmium (this toy had a reading of 6,253 ppm lead). It’s harder to tell, however, how much of any given toxicant is making its way into a child’s body.

